# EMX1 functions as a tumor inhibitor in spinal cord glioma through transcriptional suppression of WASF2 and inactivation of the Wnt/β‐catenin axis

**DOI:** 10.1002/brb3.2684

**Published:** 2022-07-18

**Authors:** Ziyin Han, Zufang Mou, Yulong Jing, Rong Jiang, Tao Sun

**Affiliations:** ^1^ Department of Traumatic Orthopedics Yantaishan Hospital of Yantai Yantai Shandong P.R. China; ^2^ Administration Department of Nosocomial Infection Yantaishan Hospital of Yantai Yantai Shandong P.R. China; ^3^ Department of Physiology Binzhou Medical University, Yantai Campus Yantai Shandong P.R. China

**Keywords:** EMX1, spinal cord glioma, transcription, WASF2, Wnt/β‐catenin

## Abstract

**Background:**

Gliomas are the most frequent and aggressive cancers in the central nervous system, and spinal cord glioma (SCG) is a rare class of the gliomas. Empty spiracles homobox genes (EMXs) have shown potential tumor suppressing roles in glioma, but the biological function of EMX1 in SCG is unclear.

**Methods:**

The EMX1 expression in clinical tissues of patients with SCG was examined. SCG cells were extracted from the tissues, and altered expression of EMX1 was then introduced to examine the role of EMX1 in cell growth and invasiveness in vitro. Xenograft tumors were induced in nude mice for in vivo validation. The targets of EXM1 were predicted via bioinformatic analysis and validated by luciferase and ChIP‐qPCR assays. Rescue experiments were conducted to validate the involvements of the downstream molecules.

**Results:**

EMX1 was poorly expressed in glioma, which was linked to decreased survival rate of patients according to the bioinformatics prediction. In clinical tissues, EMX1 was poorly expressed in SCG, especially in the high‐grade tissues. EMX1 upregulation significantly suppressed growth and metastasis of SCG cells in vitro and in vivo. EMX1 bound to the promoter of WASP family member 2 (WASF2) to suppress its transcription. Restoration of WASF2 blocked the tumor‐suppressing effect of EMX1. EMX1 suppressed Wnt/β‐catenin signaling activity by inhibiting WASF2. Coronaridine, a Wnt/β‐catenin‐specific antagonist, blocked SCG cell growth and metastasis induced by WASF2.

**Conclusion:**

This study elucidates that EMX1 functions as a tumor inhibitor in SCG by suppressing WASF2‐dependent activation of the Wnt/β‐catenin axis.

## Graphical Abstract

:This study focuses on the function of EMX1 in SCG and its related biological molecules. We first identified reduced EMX1 in SCG tissues and the isolated cells, especially in the high‐grade cancers. Upregulation of EMX1 suppressed, whereas downregulation of EMX1 promoted malignant development of SCG cells in vitro and in vivo. EMX1 suppressed the transcription of WASF2 to inactivate the Wnt/β‐catenin pathway. The tumor‐suppressive effects of EMX1 were blocked upon WASF2 restoration, and the SCG cell growth and metastasis triggered by WASF2 was suppressed by artificial Wnt/β‐catenin inhibition. Taken together, we opine that EMX1 suppresses WASF2 transcription and inactivates the Wnt/β‐catenin axis pathway to suppress SCG development.

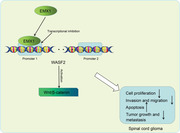

## 1 INTRODUCTION

Gliomas are the most frequent and aggressive type of primary tumors of the central nervous system (CNS) which can arise in different parts; however, most of the cases occur in the glial cells of the cerebral cortex (Tamtaji et al., 2020). Spinal cord tumors are rare relative to intracranial tumors, which account for less than 10% of all CNS neoplasms (Yang et al., 2020), and spinal cord gliomas (SCG) represent only 22% of all spinal cord tumors (Henson, 2001). According to 2016 World Health Organization (WHO) classification, gliomas are allocated into four grades (Grades I–IV): Grade I and II are nonmalignant tumors, Grade III and IV tumors are malignant, and especially the Grade IV tumors, mainly glioblastomas (GBMs), are the most aggressive and fatal one (Louis et al., 2016). Despite the major treatment options, such as surgical resection, radiotherapy, adjuvant chemotherapy, immunotherapy, targeted therapy or a combination thereof (Weller et al., 2013), the overall survival period of patients with spinal cord‐GBM was reported to be extremely short at approximately 10–14 months (Adams et al., 2012; Chanchotisatien et al., 2019). Moreover, the survivors usually have severe neurologic deficit and reduced life quality.

Empty spiracle homobox proteins (EMXs) are a class of mammalian homeobox gene transcription factors that play a critical role in neurogenesis, including neuronal motility, differentiation, and synaptic connectivity (Kobeissy et al., 2016; MuhChyi et al., 2013). EMX have two isoforms, EMX1 and EMX2, which share an array of structural and functional characteristics with overlapping patterns of spatio‐temporal expression (Chan et al., 2001; Gulisano et al., 1996; Simeone et al., 1992). Generally, they act as transcription factors in early embryogenesis and neuroembryogenesis from metazoans to higher vertebrates (Jimenez‐Garcia et al., 2021b). EMX2 has been reported as a candidate tool to suppress GBM (Falcone et al., 2016; Monnier et al., 2018). However, the role of EMX1 in glioma, especially in SCG, remains unarticulated.

The bioinformatics analysis in the present study suggested WASP family member 2 (WASF2) as a candidate target transcript of EMX1. WASF2, also known as WAVE2, is a downstream effector molecule that implicates the signal transduction from small GTPases to the actin cytoskeleton, which can elongate the cell movement mode (Yao et al., 2013). Moreover, WASF2 has been associated with the viability and radioresistance of glioma cells via the WAVE2‐Arp2/3 axis (Zhou et al., 2016). Moreover, WASF2 promoted invasiveness of cancer cells by binding to actin cytoskeletal protein alpha‐actinin 4 (ACTN4) (Taniuchi et al., 2018), and ACTN4 was associated with β‐catenin to regulate the adherence and movement of cells (Hayashida et al., 2005). Interestingly, the EMX transcription factors have been reported to suppress the Wnt/β‐catenin pathway for tumor inhibition (Jimenez‐Garcia et al., 2021a). Taken together, this study was conducted to validate the potential interactions between EMX1, WASF2, and β‐catenin and their roles in SCG development.

## 2 MATERIALS AND METHODS

### 2.1 Clinical samples

Ten patients with SCG treated at Yantaishan Hospital of Yantai from 2019 to 2020 were included in the study. The tumor and adjacent tissue samples were collected by surgery. All patients had complete clinic information, and they neither had other malignancies nor had a history of chemo‐ or radio‐therapies. This research was approved by the Ethics Committee of Yantaishan Hospital of Yantai and adhered to the *Helsinki Declaration*. All patients signed the informed consent form.

According to the 2016 WHO Classification of Tumors of the CNS (Louis et al., 2016), the patients were allocated into high‐grade SCG (H‐SCG, WHO III and WHO IV) and low‐grade SCG (L‐SCG, WHO Ⅱ and WHO Ⅰ). The detailed information is given in Table 1.

**TABLE 1:** Clinical features of patients with H‐SCG or L‐SCG

Patient ID	Age (years)	Sex	Tumor pathology classification	Tumor grade
4039959	15	Male	Glioblastoma	WHO IV
4315021	46	Male	Diffuse midline glioma (H3 K27M–mutant)	WHO IV
3905157	11	Female	Diffuse midline glioma (H3 K27M–mutant)	WHO IV
4373834	16	Female	Anaplastic astrocytomas	WHO III
4443282	27	Male	Anaplastic pleomorphic xanthoastrocytoma	WHO III
3992882	18	Female	Diffuse astrocytomas	WHO II
2992826	13	Female	Diffuse astrocytomas	WHO II
4101703	35	Male	Pleomorphic xanthoastrocytomas	WHO II
3037960	24	Male	Pilocytic astrocytoma	WHO I
2594576	15	Male	Pilocytic astrocytoma	WHO I

*Note*: H‐SCG, high‐grade spinal cord glioma; L‐SCG, low‐grade spinal cord glioma.

### 2.2 Cell isolation and culture

SCG cells were isolated as previously reported (Xu et al., 2021). Fresh human SCG tissues were cut into 1‐mm^3^ fragments and washed in phosphate‐buffered saline (PBS). Next, 1% penicillin and streptomycin solution (Gibco; Thermo Fisher Scientific Inc., Waltham, MA, USA), and the tissue fragments were digested in 0.25% trypsin (Gibco) in water bath at 37°C for 30 min. The cell fluid was filtered with a 100‐mesh steel filter to produce tumor cell suspension. The suspension was loaded into tubes for 10 min of centrifugation at 300 g at 25°C. After that, the cells were resuspended in Roswell Park Memorial Institute‐1640 (Gibco) supplemented with 10% fetal bovine serum (FBS; Gibco) and 1% penicillin and streptomycin and cultured in a 37°C incubator with 5% CO_2_. Cells isolated from the tissue sample having the lowest EMX1 expression (Patient ID: 4039959) or the highest (Patient ID: 3037960) were named H‐SCG and L‐SCG, respectively

### 2.3 Cell transfection and treatment

Mammalian gene expression vector‐based DNA overexpression vectors including Vector‐EMX1 and Vector WASF2 were procured from VectorBuilder (Guangzhou, Guangdong, China). Vector‐NC (negative control) was used as control. Short hairpin RNA (shRNA) of EMX1 and WASF2 (sh‐EMX1 1, 2, 3#; sh‐WASF2 1, 2, 3#) and the control sh‐NC were procured from RiboBio Co., Ltd (Guangzhou, Guangdong China). All vectors or shRNAs were transfected into cells in accordance with the instructions of the Lipofectamine2000 kit (Thermo Fisher Scientific).

A Wnt/β‐catenin‐specific antagonist Coronaridine (Cat. No. HY‐121118; MedChemExpress, Monmouth Junction, NJ, USA) was used to treat the cells at 50 μM to specifically suppress the Wnt/β‐catenin activity (Ohishi et al., 2015). Cells treated with dimethyl sulphoxide (DMSO) were set as controls.

### 2.4 Reverse transcription quantitative polymerase chain reaction

Total RNA from cells or tissues was isolated using TRIzol (Takara Holdings Inc., Kyoto, Japan) and reverse‐transcribed to cDNA using a reverse transcription kit (Takara). The cDNA was used for reverse transcription quantitative polymerase chain reaction (qPCR) using the TB Green^®^ real‐time PCR kit (Takara) on a Bio‐Rad CFX96 system (Bio‐Rad, Hercules, CA, USA). Fold change in gene expression was measured using the 2^–ΔΔCt^ method. The primers are presented in Table 2, in which GAPDH was the endogenous loading.

**TABLE 2:** Primers for RT‐qPCR

Gene symbol	Forward primer(5ʹ−3ʹ)	Reverse primer(5ʹ−3ʹ)
EMX1	GCCTTCGAGAAGAACCACTACG	CGGTTCTGGAACCACACCTTCA
WASF2	CACCACAGTCAGACTCTGCTTC	CCAGATCCTCTTTGGTTGTCCAC
β‐catenin	CACAAGCAGAGTGCTGAAGGTG	GATTCCTGAGAGTCCAAAGACAG
GAPDH	GTCTCCTCTGACTTCAACAGCG	ACCACCCTGTTGCTGTAGCCAA

*Note*: RT‐qPCR, reverse transcription quantitative polymerase chain reaction; EMX1, empty spiracles homeobox 1; WASF2, WASP family member 2.

### 2.5 Western blot analysis

Cells were fully mixed with the radio‐immunoprecipitation assay lysis buffer (Beyotime Biotechnology Co., Ltd., Shanghai, China) at 4°C to extract total protein. The supernatant was collected after centrifugation at 12,000 rpm. The protein sample was separated by SDS‐PAGE and transferred onto polyvinylidene difluoride membranes (Thermo Fisher Scientific). The membranes were blocked by 5% nonfat milk for 2 h, incubated with the diluted primary antibodies (Table 3) overnight at 4°C, and then with goat anti‐rabbit IgG H&L (HRP) (1:10,000; ab6721; Abcam Inc., Cambridge, MA, USA) at 25°C for 2 h. The blot bands were visualized using the enhanced chemiluminescence system (GE Healthcare, Chicago, IL, USA). Relative protein expression was examined using the Image J (NIH, Bethesda, MD, USA). GAPDH was used as the endogenous control.

**TABLE 3:** Primary antibodies for western blot analysis

Antibodies	Dilution	It. No	Manufacture
EMX1	1:1,000	PA5‐101636	Thermo Fisher Scientific
WASF2	1:1,000	#3659	Cell Signaling Technology
β‐catenin	1:5,000	ab32572	Abcam
GAPDH	1:10,000	ab181602	Abcam

*Note*: EMX1, empty spiracles homeobox 1; WASF2, WASP family member 2; GAPDH, glyceraldehyde‐3‐phosphate dehydrogenase; Thermo Fisher Scientific Inc., Waltham, MA, USA; Cell Signaling Technology, Beverly, MA, USA; Abcam Inc., Cambridge, MA, USA.

### 2.6 Cell counting kit‐8 method

Cell proliferation was evaluated using a cell‐counting kit‐8 (CCK‐8 kit) (Abcam). The cells were cultured in 96‐well plates at 1 × 10^4^ cells/well. At the indicated time points (0, 24, 48, and 72 h), each well was filled with 10 μl CCK‐8 reagent for another 2 h of incubation at 37°C. The optical density (OD) at 460 nm was examined using a microplate reader.

### 2.7 5‐ethynyl‐2′‐deoxyuridine (EdU) labeling assay

A 5‐ethynyl‐2′‐deoxyuridine (EdU) labeling kit (Beyotime) was used to evaluate DNA replication activity of cells. The cells were seeded in 96‐well plates at 1 × 10^5^ cells/well, fixed in 4% paraformaldehyde (PFA) for 30 min, and incubated with 2 mg/ml glycine for 5 min to neutralize the remaining PFA. Thereafter, the cells were permeabilized by 0.5% Triton X‐100 for 20 min. Each well was added with the Click‐iT reaction mixture for 30 min of incubation in the dark at 25°C. After that, the cells were stained with Hoechst 33342 solution in the dark for 30 min. The labeling was detected under a fluorescence microscope (Carl Zeiss, Oberkochen, Germany), and the EdU‐positive cells were quantified using the Image J software.

### 2.8 Wound‐healing assay

The SCG cells were incubated in 6‐well plates at 1 × 10^4^ cells/well. When the confluence reached 80–90%, a pipette tip was used to scratch the monolayer cells. The cell debris was washed away with PBS, and the remaining cells were cultured in fresh FBS‐free medium at 37°C. The width of the scratches at 0 and 24 h was captured using an optical microscope (Zeiss). The percentage of migrated area was measured using the Image J.

### 2.9 Transwell assay

Invasion of cells was determined using Matrigel (BD Biosciences, Shanghai, China)‐precoated Transwell chambers (Corning Glass Works, Corning, NY, USA). Cells (2 × 10^4^) in serum‐free medium was loaded in the apical chambers, and the basolateral chambers were added with 10% FBS‐contained medium for inducer. After 48 h, the cells invaded to the lower membrane were fixed in PFA (Solarbio Science & Technology Co., Ltd., Beijing, China) and stained with crystal violet. The number of invaded cells was counted under the microscope with five random fields included.

### 2.10 Luciferase assay

The WASF2 promoter sequence containing the putative binding site with EMX1 was obtained from the UCSC system (https://genome.ucsc.edu/). The sequence was inserted into the pGL3‐Basic vector (Promega Corporation, Madison, WI, USA) to construct luciferase reporter vectors. The Promoter vector was co‐transfected with Vector‐EMX1 and the control into H‐SCG cells (1 × 10^6^), or co‐transfected with sh‐EMX1 and the control into L‐SCG cells (1 × 10^6^) using Lipofectamine 2000. After 48 h, the luciferase activity in cells was examined using a dual luciferase reporter system (Promega).

A Transfection grade T‐cell factor (TCF) Reporter Plasmid Kit (Sigma‐Aldrich; Merck KGaA, Darmstadt, Germany) was used for to examine the activity of the Wnt/β‐catenin signaling by the TOP/FOP flash assay. The treated SCG cells were cultured on 6‐well plates at 1 × 10^6^ cells per well. The TOP, FOP and PRL plasmids were transfected into cells using FuGENE 6 (Promega). The luciferase activity in cells was measured 48 h later.

### 2.11 Chromatin immunoprecipitation (ChIP)‐qPCR

A Pierce agarose ChIP kit (Thermo Fisher Scientific) was used to examine the enrichment of WASF2 promoter by EMX1. In short, the SCG cells (1 × 10^6^) were fixed in 4% PFA for 10 min and then treated with glycine to terminate the reaction. The cells were lysed in protease/phosphatase inhibitor‐contained lysis buffer on ice and the centrifuged at 9,000 *g* for 10 min to collect the supernatant‐containing chromatin. The supernatant was reacted with anti‐EMX1 (sc‐398115, Santa Cruz Biotechnology, Inc, Santa Cruz, CA, USA) at 4°C overnight for immunoprecipitation, normal rabbit IgG (Thermo Fisher Scientific) was used as control. After that, the antibody was captured by ChIP Grade Protein A/G Plus Agarose, and the complexes were eluted using IP elution buffer. The DNA binding buffer, DNA column wash buffer, and DNA column elution solution were used for DNA retrieval and purification. The abundance of WASF2 promoter in DNA was analyzed by qPCR. The primer sequences were as follows: Promoter 1#: Forward primer: 5ʹ‐GACTTGTTGCTCACCTGGC‐3ʹ, Reverse primer: 5ʹ‐GCGTAATGGCGGACACAG‐3ʹ; Promoter 2#: Forward primer: 5ʹ‐ GGGTGCAAACCAAGTGAAGT‐3ʹ, Reverse primer: 5ʹ‐TGGCCCCTTCCTTTCACTAA‐3ʹ

### 2.12 Tumor growth and metastasis in nude mice

Sixty 4‐week‐old female BALB/c nude mice were procured from Vital River Laboratory Animal Technology Co., Ltd. (Beijing, China). All mice were cultured in specific‐pathogen‐free (SPF) grade condition with free access to feed and water. All animal procedures were approved by the Animal Ethics Committee of Yantaishan Hospital of Yantai and performed according to the Guide for the Care and Use of Laboratory Animals (NIH). According to the differences in the treatment of H‐SCG cells, the nude mice were allocated into six groups: Vector‐NC, Vector‐EMX1, Vector‐EMX1 + Vector‐NC, Vector‐EMX1 + Vector‐WASF2, Vector‐EMX1 + Vector‐WASF2 + DMSO, and Vector‐EMX1 + Vector‐WASF2 + Coronaridine groups, *n* = 10 in each group. Five mice were used to evaluate tumor growth, and the rest five mice were used to evaluate tumor metastasis in vivo.

For tumor growth measurement, stably transfected H‐SCG cells (1 × 10^5^) were injected into the mice through subcutaneous injection at the left abdomen. The volume of the tumors was determined once a week as follows: Volume = Length × Width^2^/2. After 4 weeks, the mice were euthanized via injection of 150 mg/kg pentobarbital sodium, and the xenograft tumors were collected and weighed.

For tumor metastasis measurement, stably transfected H‐SCG cells (1 × 10^6^) were injected into the mice through tail vein injection. After 5 weeks, the mice were euthanized, and the lung tissues were collected to examine tumor infiltration via the hematoxylin and eosin (HE) staining.

### 2.13 HE staining

The clinical SCG tissues or mouse lung tissues were embedded in paraffin and cut into 5‐μM slices. The slices were dewaxed and rehydrated. The slices were stained with hematoxylin (Sigma‐Aldrich) for 5 min, differentiated in 1% HCl‐ethanol for 3 s, rinsed with distilled water, and stained with eosin (Sigma) for 3 min. The slices were then dehydrated in ethanol, cleared in xylene, and sealed by neutral balsam for observation under the microscope. The tumor infiltration in lung tissues was quantified using the Image J software.

### 2.14 Statistical analysis

All data analyses were performed by Prism 8.02 (GraphPad, La Jolla, CA, USA). Data were collected expressed as the mean ± standard deviation (SD) from three independent experiments. The paired or unpaired *t* test was used for the comparison between two groups, and one‐ or two‐way analysis of variance (ANOVA) was used for the comparison among multiple groups, followed by Tukey's‐ post hoc test. Correlation between variables was analyzed by the Pearson's correlation analysis. *p < .05 was considered to show statistical significance.

## 3 RESULTS

### 3.1 Poor expression of EMX1 in SCG is correlated with advanced tumor grades

**FIGURE 1:**
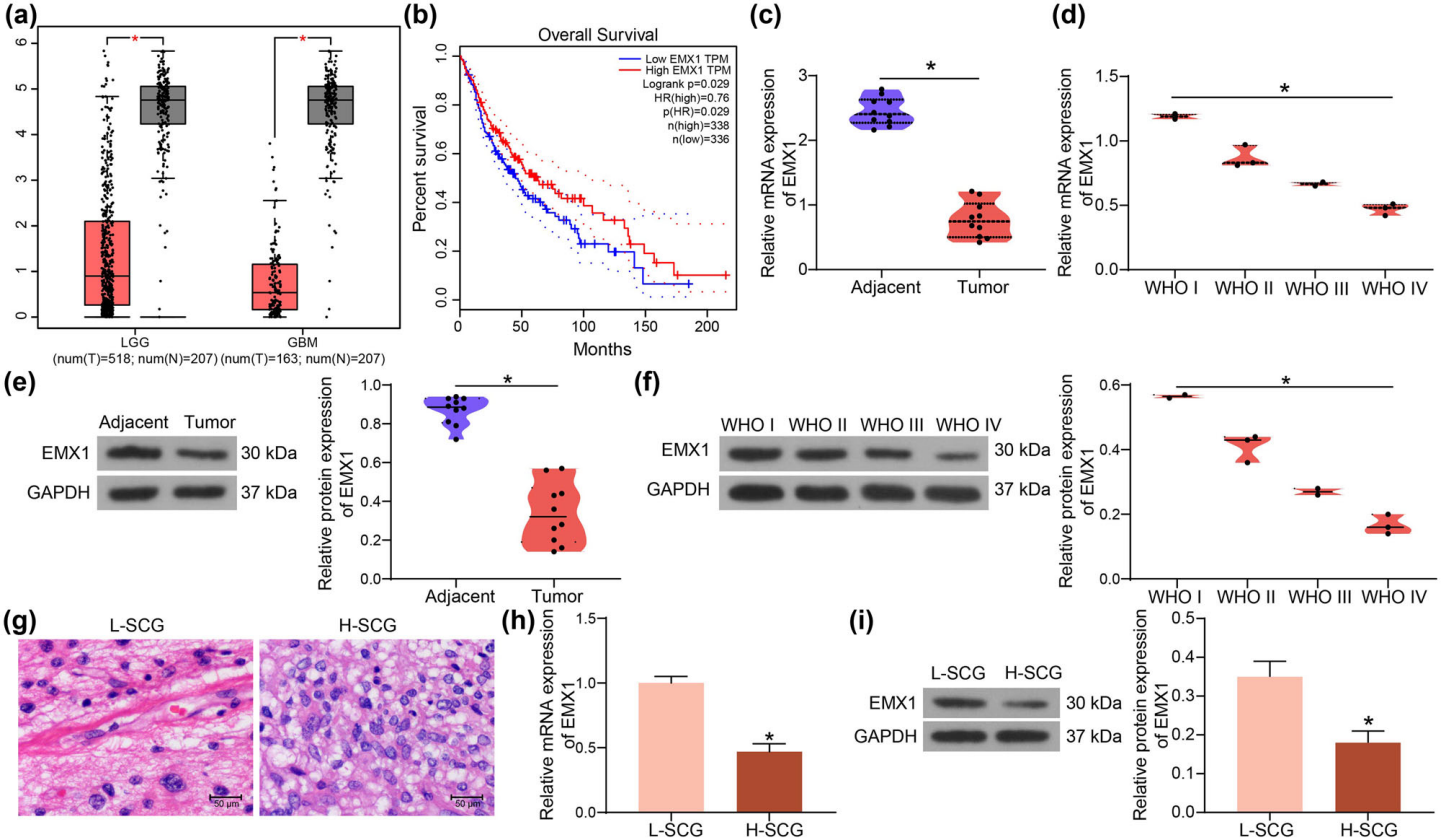
Poor expression of EMX1 in SCG is correlated with increased tumor grades. (a) expression of EMX1 in glioma patients in the GEPIA database; (b) correlation between EMX1 expression and the overall survival of glioma patients in the GEPIA database; (c) expression of EMX1 mRNA in the SCG tissues and the adjacent normal tissues examined by RT‐qPCR; (d) expression of EMX1 mRNA in different grades of SCG; (e) expression of EMX1 protein in the SCG tissues and the adjacent normal tissues examined by western blot analysis; (f) expression of EMX1 protein in different grades of SCG; (g) histological characteristics of L‐SCG and H‐SCG tissues observed by HE staining; H‐I, mRNA (h) and protein (i) levels of EMX1 in L‐SCG and H‐SCG cells detected by RT‐qPCR and western blot assays. Three repetitions were performed for cellular experiments. Differences were analyzed by the paired *t* test (c and e), the unpaired *t* test (h and i), and one‐way ANOVA (d and f). *p < .05

Before the examination of the role of EMX1 in SCG, its expression profile was first predicted in the GEPIA system (http://gepia.cancer‐pku.cn/index.html). Data in the bioinformatics system showed that the expression of EMX1 was significantly reduced in glioma (Figure 1a), and patients with high EMX1 expression had greater overall survival rate than those with low EMX1 expression (Figure 1b). To validate this, we examined the expression of EMX1 in SCG. The RT‐qPCR results indicated that the EMX1 expression was reduced in SCG tissues versus that in the adjacent normal tissues (Figure 1c), and it was reduced as the tumor grade increases (Figure 1d) (WHO I vs. WHO II: *p* = .0040; WHO I vs. WHO III: *p* = .0005; WHO I vs. WHO IV: *p* < .0001; WHO II vs. WHO III: *p* = .0330; WHO II vs. WHO IV: *p* = .0007; WHO III vs. WHO IV: *p* = .0407). Similarly, the protein level of EMX1 was decreased in SCG tissues (Figure 1e), and it also showed a decreasing trend as the tumor grade increases (Figure 1f) (WHO I vs WHO II: *p* = .0066; WHO I vs. WHO III: *p* = .0003; WHO I vs. WHO IV: *p* < .0001; WHO II vs. WHO III: *p* = .0109; WHO II vs. WHO IV: *p* = .0003; WHO III vs. WHO IV: *p* = .0424).

Thereafter, the SCG tissue samples with the lowest EMX1 expression (Patient ID: 4039959) and the highest EMX1 expression (Patient ID: 3037960) were used for the extraction of H‐SCG and L‐SCG cells, respectively. The histological characteristics of these two samples were observed by Hematoxylin and eosin (HE) staining. The dense areas of the L‐SCG tissues contained Rosenthal fibers, whereas the loose areas had bipolar cells surrounding the blood vessels. The H‐SCG tissues showed a diffused distribution of cells with significant nuclear atypia and vascular hyperplasia (Figure 1g). RT‐qPCR and western blot assays indicated that the expression of EMX1 was significantly lower in H‐SCG cells than that in L‐SCG cells (Figure 1h,i).

### 3.2 EMX1 overexpression suppresses growth and metastasis of SCG cells

**FIGURE 2:**
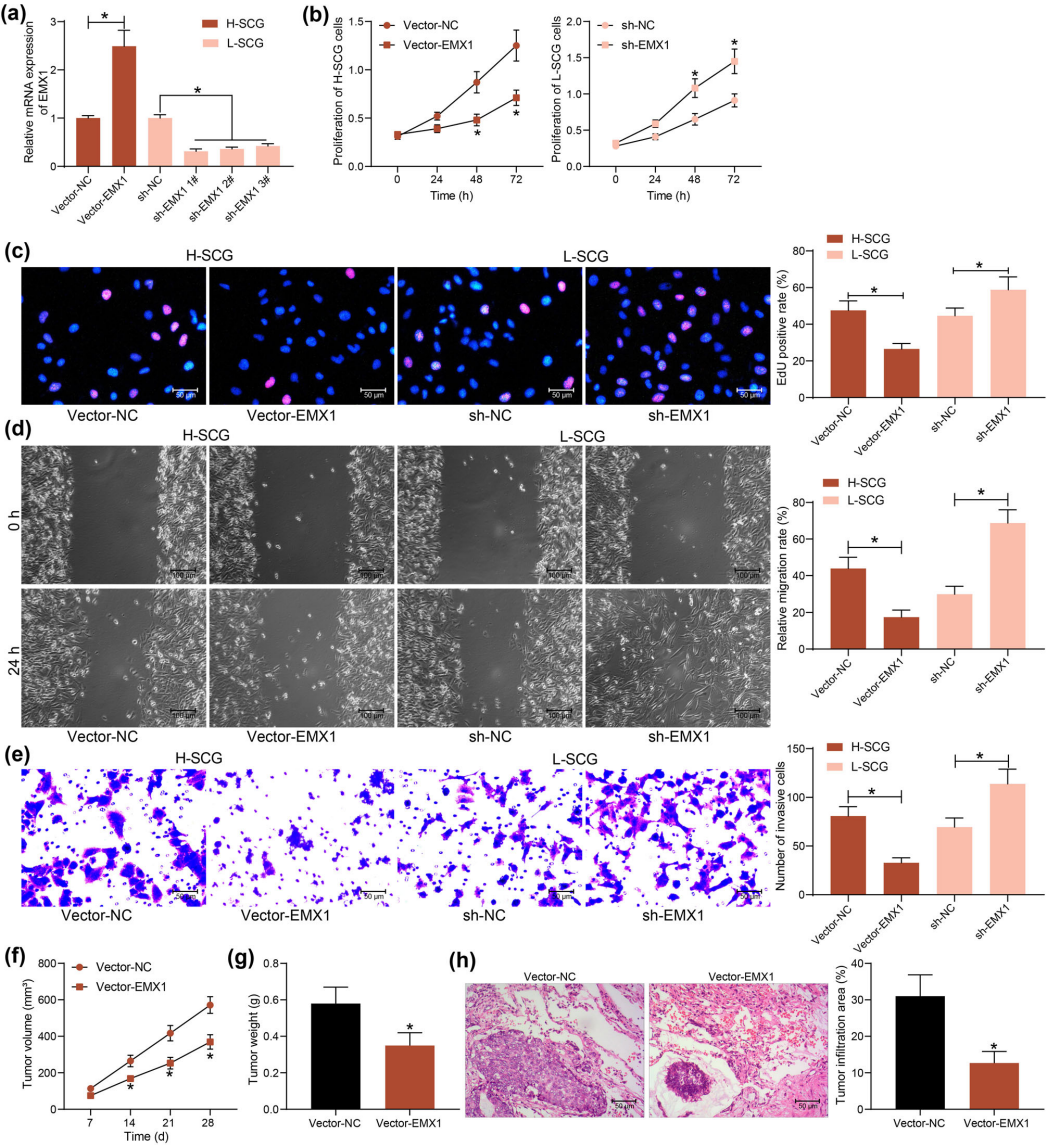
EMX1 overexpression suppresses growth and metastasis of SCG. (a) transfection efficiency of Vector‐EMX1 and shRNAs (sh‐EMX1 1, 2, 3#) in SCG cells examined by RT‐qPCR; (b) proliferation of the SCG cells examined by the CCK‐8 method; (c) DNA replication ability of the SCG cells determined by the EdU labeling assay; migratory (d) and invasive (e) activities of the SCG cells examined by wound‐healing and Transwell assays, respectively; growth rate (f) and the weight on the 28th day (g) of the xenograft tumors in nude mice in the setting of subcutaneous injection of H‐SCG cells; (h) tumor cell infiltration in mouse lung tissues at 5 weeks after tail vein injection of H‐SCG cells examined by HE staining. Three repetitions were performed for cellular experiments. In animal experiments, *n* = 5 in each group. Differences were analyzed by the unpaired *t* test (g and h), one‐way ANOVA (a, c, d, and e) and two‐way ANOVA (b and f). *p < .05

The role of EMX1 in the growth of SCG cells was first examined in vitro. The Vector‐EMX1 overexpressing EMX1 and the control vector were transfected into H‐SCG cells, and the shRNAs (sh‐EMX1 1, 2, 3#) were transfected into L‐SCG cells. Vector‐EMX1 significantly elevated EMX1 expression in H‐SCG cells, and the three shRNAs sh‐EMX1 1, 2, 3# reduced EMX1 expression in L‐SCG cells (Figure 2a). The sh‐EMX1 1# with the best interfering efficiency was selected for subsequent use.

The CCK‐8 method was then performed to examine the proliferation of cells. Overexpression of EMX1 in SCG cells suppressed cell proliferation, whereas downregulation of EMX1 in L‐SCG cells promoted cell proliferation (Figure 2b). In line, the EdU labeling assay suggested that the DNA replication of cells was suppressed after EMX1 overexpression but increased following EMX1 silencing (Figure 2c). The wound‐healing and Transwell assays showed that the migratory and invasive activities of H‐SCG cells were blocked by Vector‐EMX1, but inverse results were identified in L‐SCG cells with EMX1 silencing (Figure 2d,e).

Cells were injected into nude mice for in vivo experiments. To avoid unnecessary animal death, only H‐SCG cells stably transfected with Vector‐EMX1 were used. Overexpression of EMX1 significantly slowed down the growth rate of xenograft tumors in mice (Figure 2f) and reduced the tumor weight (Figure 2g). Moreover, EMX1 overexpression also significantly reduced the size of tumor cell infiltration in mouse lung tissues in the setting of tail vein injection (Figure 2h).

### 3.3 WASF2 is a candidate target of EMX1 in SCG

**FIGURE 3:**
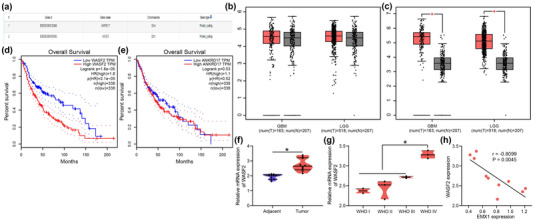
WASF2 is a candidate target of EMX1 in SCG. (a) candidate downstream targets of EMX1 predicted in the hTFtarget system; expression of ANKRD17 (b) and WASF2 (c) in glioma patients in the GEPIA database; correlations of the WASF2 (d) and ANKRD17 (e) with the overall survival of glioma patients in the GEPIA database; (f) expression of WASF2 mRNA in the SCG tissues and the adjacent normal tissues examined by RT‐qPCR; (g) expression of EMX1 in different grades of SCG; (h) an inverse correlation between WASF2 and EMX1 in SCG tissues. Three repetitions were performed. Differences were analyzed by the paired *t* test (f) and one‐way ANOVA (g). *p < .05

EMX1 can function as a transcription factor to suppress cancer progression (Jimenez‐Garcia et al., 2021b). Therefore, the potential downstream targets of EMX1 were predicted in the hTFtarget system (http://bioinfo.life.hust.edu.cn/hTFtarget/#!/), and two targets were predicted: ANKRD17 and WASF2 (Figure 3a). The expression of ANKRD17 (Figure 3b) and WASF2 (Figure 3c) in glioma was predicted in the GEPIA system. WASF2 was suggested to be highly expressed in glioma, whereas the ANKRD17 expression was not significantly changed in glioma tissues compared to normal tissues. In addition, low expression of WASF2 in glioma was correlated with increased survival rate (Figure 3d), whereas the ANKRD17 expression was suggested to have little effect on the survival rate of patients (Figure 3e).

Therefore, WASF2 was selected for the subsequent research. RT‐qPCR results indicated that WASF2 was upregulated in the collected SCG tissues compared to the adjacent normal tissues (Figure 3f). In addition, SCG tissues with higher WHO grades showed higher WASF2 expression (Figure 3g). The WASF2 expression in SCG tissues showed an inverse correlation with EMX1 expression (Figure 3h).

### 3.4 EMX1 transcriptionally suppresses WASF2 expression

**FIGURE 4:**
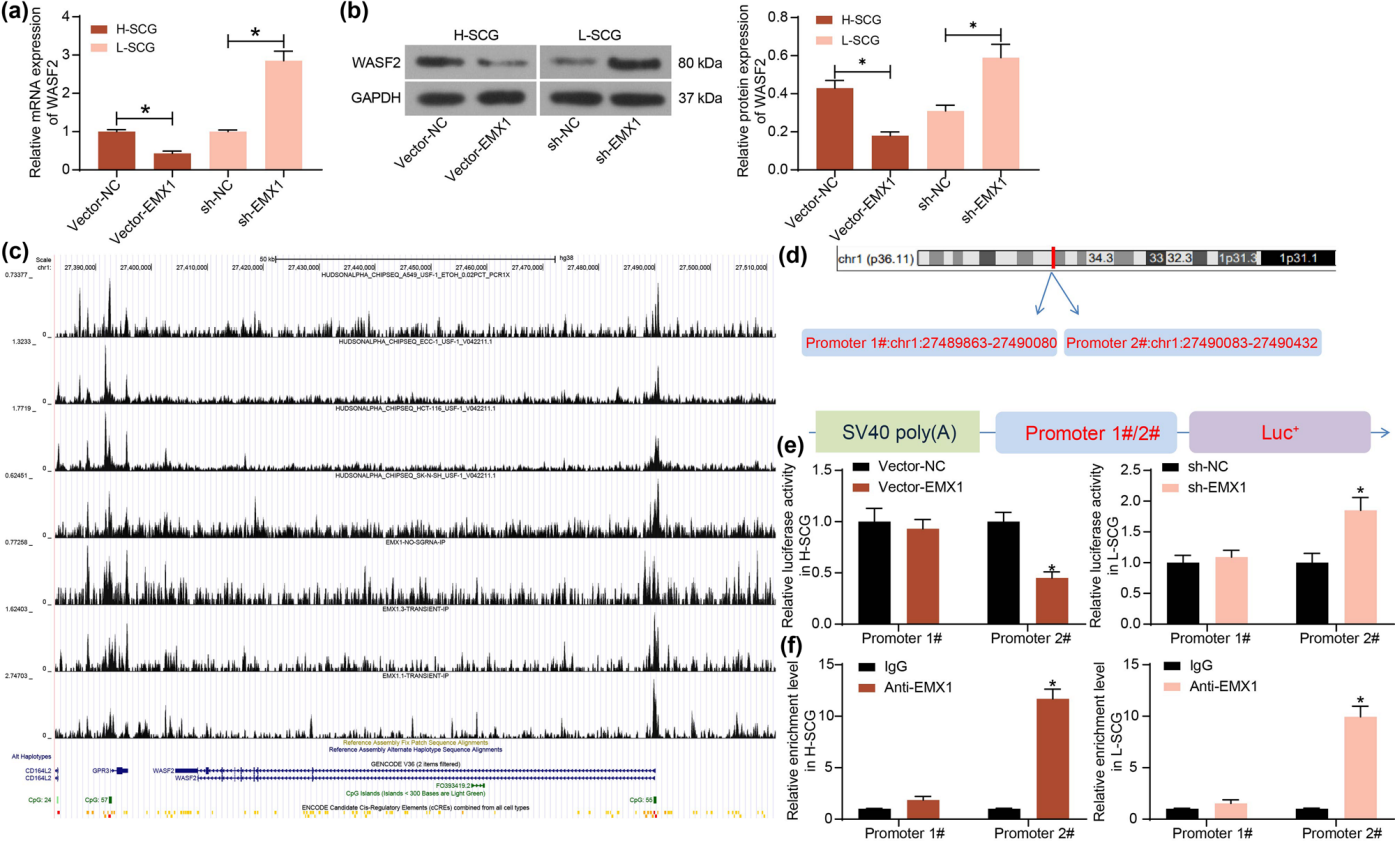
EMX1 transcriptionally suppresses WASF2 expression. mRNA (a) and protein (b) levels of WASF2 in L‐SCG and H‐SCG cells with altered EMX1 expression detected by RT‐qPCR and western blot assays; (c) potential regulatory site of EMX1 on WASF2 predicted using the Cistrome Data Browser; (d) the Promoter 1# and Promoter 2# sequences for the construction of luciferase reporter vectors; (e) effect of EMX1 on the activity of WASF2 promoter examined by the dual luciferase reporter gene assay; (f) enrichment of Promoter 1# and Promoter 2# sequences by anti‐EMX1 examined by the ChIP‐qPCR assay. Three repetitions were performed for cellular experiments. Differences were analyzed by the one‐way ANOVA (a and b) or two‐way ANOVA (e and f). *p < .05

To confirm the regulation of EMX1 on WASF2, the expression of WASF2 in SCG after EXM1 inference was examined. Of note, overexpression of EMX1 led to a decline in WASF2 expression in H‐SCG cells, whereas silencing of EMX1 led to an increase in WASF2 expression in L‐SCG cells (Figure 4a,b).

The regulatory site of EMX1 on WASF2 was predicted using the Cistrome Data Browser (http://cistrome.org/db/#/) (Figure 4c). It was observed that EMX1 was enriched surrounding the WASF2 promoter, where two promoter sites exist, which are the potential binding sites for EMX1 (Figure 4d). The two potential binding sites were named Promoter 1# and Promoter 2# and inserted into the luciferase reporter vectors to construct reporter vectors (Figure 4d) for luciferase assay. It was observed that the Vector‐EMX1 significantly suppressed the luciferase activity of Promoter 2# whereas sh‐EMX1 significantly enhanced the luciferase activity of Promoter 2# in SCG cells. But alteration of EMX1 did not affect the luciferase activity of Promoter 1# (Figure 4e). Moreover, the ChIP‐qPCR assay indicated that an abundance of Promoter 2# sequence was enriched by anti‐EMX1 compared to IgG (Figure 4f). However, anti‐EMX1 did not enrich the sequence of Promoter 1#. These results confirmed that EMX1 binds to the WASF2 promoter (chr1: 27490083–27490432) to suppress its transcription.

### 3.5 WASF2 affects the tumor‐inhibiting role of EMX1

**FIGURE 5:**
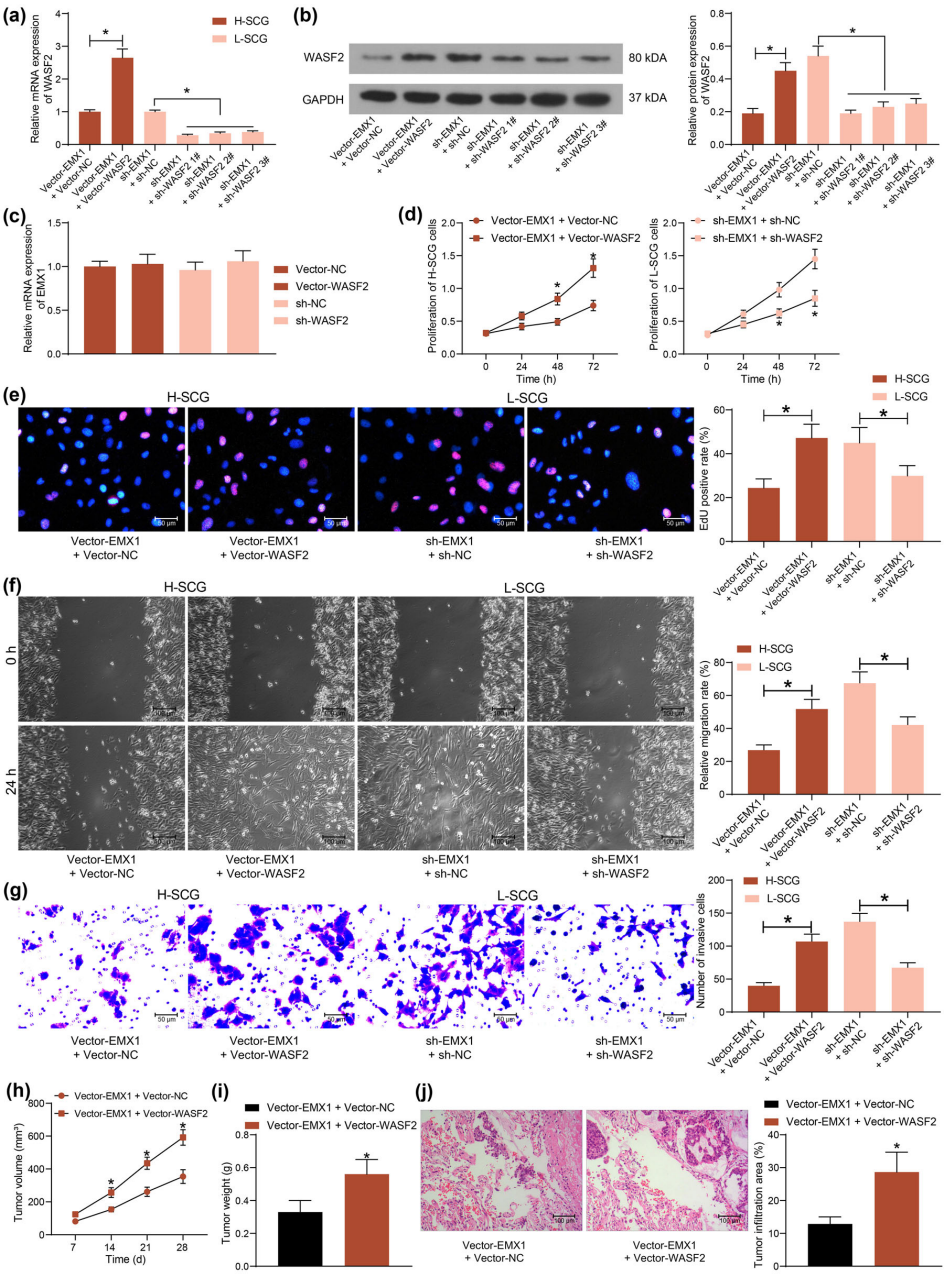
WASF2 affects the tumor‐inhibiting role of EMX1. (a) transfection efficiency of Vector‐WASF2 and sh‐WASF2 1, 2, 3# in SCG cells determined by RT‐qPCR; (b) protein expression of EMX1 in SCG cells after Vector‐WASF2 or sh‐WASF2 1, 2, 3# transfection determined by western blot analysis; (c) mRNA expression of EMX1 in SCG cells after separate transfection of Vector‐WASF2 or sh‐WASF2 in cells; (d) proliferation of the SCG cells examined by the CCK‐8 method; (e) DNA replication ability of the SCG cells determined by the EdU labeling assay; f‐g, migratory (f) and invasive (g) activities of the SCG cells examined by wound‐healing and Transwell assays, respectively; h‐i, growth rate (h) and the weight on the 28th day (i) of the xenograft tumors in nude mice in the setting of subcutaneous injection of H‐SCG cells; j, tumor cell infiltration in mouse lung tissues at 5 weeks after tail vein injection of H‐SCG cells examined by HE staining. Three repetitions were performed for cellular experiments. In animal experiments, *n* = 5 in each group. Differences were analyzed by the unpaired *t* test (i and j), one‐way ANOVA (a, b, c, f, and g) and two‐way ANOVA (d and h). *p < .05

To confirm the interaction between EMX1 and WASF2, Vector‐WASF2 was further transfected into H‐SCG cells after Vector‐EMX1 transfection, whereas shRNA of WASF2 (sh‐WASF2 1, 2, 3#) were transfected into L‐SCG cells after sh‐EMX1 transfection. Vector‐WASF2 significantly restored the expression of WASF2 mRNA and protein in H‐SCG cells, and sh‐WASF2 1, 2, 3# reduced the WASF2 mRNA and protein levels in L‐SCG cells (Figure 5a,b). The sh‐WASF2 1# with the best interfering efficiency was collected for subsequent use. Also, Vector‐WASF2 or sh‐WASF2 was separately transfected into L‐SCG and H‐SCG cells. It was found that the transfection of Vector‐WASF2 or sh‐WASF2 alone did not affect the mRNA expression of EMX1 in cells (Figure 5c).

Thereafter, the proliferation of cells was examined. Restoration of WASF2 in SCG cells recovered the proliferation of H‐SCG cells suppressed by EMX1, whereas silencing of WASF2 blocked the proliferation of sh‐EMX1‐transfected L‐SCG cells (Figure 5D). The EdU labeling assay also indicated that DNA replication ability of SCG cells was rescued by WASF2 overexpression but reduced after WASF2 silencing (Figure 5e). The migration and invasion of SCG cells, according to the wound‐healing and Transwell assays, was promoted by WASF2 but suppressed by EMX1 (Figure 5f,g).

In vivo, further overexpression of WASF2 in SCG cells increased the growth rate (Figure 5h) and weight on the 28th day (Figure 5i) of the xenograft tumors in nude mice. In addition, overexpression of WASF2 also reduced the size or tumor infiltration in mouse lung tissues after the tail vein injection of the SCG cells (Figure 5j).

### 3.6 EMX1 suppresses WASF2 transcription to affect the transduction of the Wnt/β‐catenin signaling

**FIGURE 6:**
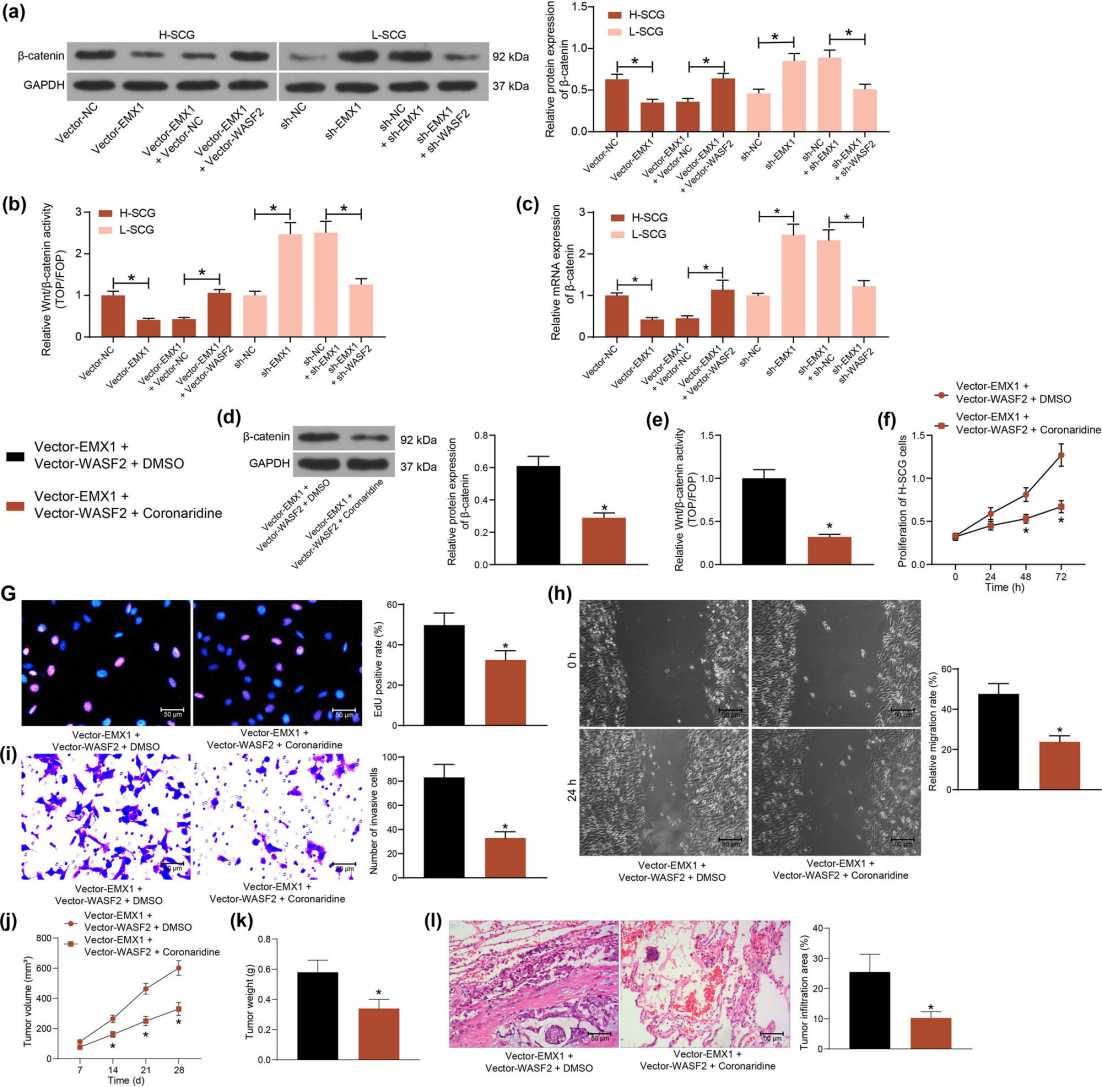
EMX1 suppresses WASF2 transcription to affect the transduction of the Wnt/β‐catenin signaling. (a) protein level of β‐catenin in SCG cells after EMX1/WASF2 alterations examined by western blot analysis; (b) activity of Wnt/β‐catenin in cells after EMX1/WASF2 alterations determined by the TOP/FOP flash assay; (c) mRNA expression of β‐catenin in SCG cells after EMX1/WASF2 alterations examined by RT‐qPCR; (d) protein level of β‐catenin in SCG cells after further Coronaridine treatment determined by western blot analysis; (e) activity of Wnt/β‐catenin in SCG cells after further Coronaridine treatment determined by the TOP/FOP flash assay; (f) proliferation of the SCG cells examined by the CCK‐8 method; (g) DNA replication ability of the SCG cells determined by the EdU labeling assay; migratory (h) and invasive (i) activities of the SCG cells examined by wound‐healing and Transwell assays, respectively; j‐k, growth rate (j) and the weight on the 28th day (k) of the xenograft tumors in nude mice in the setting of subcutaneous injection of H‐SCG cells; (l) tumor cell infiltration in mouse lung tissues at 5 weeks after tail vein injection of H‐SCG cells examined by HE staining. Three repetitions were performed for cellular experiments. In animal experiments, *n* = 5 in each group. Differences were analyzed by the unpaired *t* test (d, e, g–i, k, and l), one‐way ANOVA (a, b and c) and two‐way ANOVA (f and j). *p < .05

Inhibition of the Wnt/β‐catenin signaling has been demonstrated to be implicated in the tumor‐suppressive effect of EMX proteins (Jimenez‐Garcia et al., 2021a). Therefore, the activity of the Wnt/β‐catenin signaling in SCG cells was determined. The western blot analysis indicated that the protein level of β‐catenin in cells was suppressed by EMX1 overexpression but increased after EMX1 silencing. But the changes in β‐catenin level mediated by EMX1 were counteracted by WASF2 (Figure 6a). Similar trends were found by the TOP/FOP flash assay that EMX1 significantly suppressed the activity of the Wnt/β‐catenin signaling by inhibiting WASF2 expression (Figure 6b). Likewise, the RT‐qPCR assay revealed that the mRNA expression of β‐catenin in cells was significantly suppressed by Vector EMX1 but enhanced by sh‐EMX1, or increased by further overexpression of WASF2 or suppressed by WASF2 knockdown (Figure 6c).

The findings above suggested that restoration of WASF2 blocked the tumor‐suppressing role of EMX1 in SCG. To examine the potential involvement of the Wnt/β‐catenin axis in the process, a Wnt/β‐catenin‐specific antagonist Coronaridine was further used to treat the H‐SCG cells following Vector‐EMX1 + Vector‐WASF2 transfection. It was found that the protein level of β‐catenin and the Wnt/β‐catenin activity restored by WASF2 were significantly suppressed by Coronaridine (Figure 6d). Likewise, the TOP/FOP flash assay suggested that the activity of Wnt/β‐catenin was suppressed by Wnt/β‐catenin (Figure 6e).

In this setting, the malignant behaviors of SCG cells were examined. Inhibition of the Wnt/β‐catenin significantly suppressed WASF2‐promoted proliferation of cells (Figure 6f). The Coronaridine treatment also suppressed the DNA replication (Figure 6g), migration (Figure 6h) and invasion (Figure 6i) abilities of the SCG cells.

In vivo, Coronaridine‐mediated Wnt/β‐catenin inhibition blocked the growth rate (Figure 6j) and weight on the 28th day (Figure 6k) of the xenograft tumors in mice. Also, Wnt/β‐catenin inhibition significantly reduced the tumor cell infiltration in mouse lung tissues (Figure 6l).

## 4 DISCUSSION

Surgery is primarily the most important for the treatment of glioma, but SCGs are usually inoperable considering possible side effects. Therefore, radiotherapy is primarily considered instead in many cases, but there is still controversy for the optimal radiotherapy strategies (Choi et al., 2019). Identifying novel molecular tools or targets is promising to develop more ideas for the management of SCG. In the present paper, the authors report a novel EMX1/WASF2/Wnt/β‐catenin axis that is potentially implicated in the development of high‐grade SCG.

Owing to the lack of samples, the largest single‐organization study to date, to the best of our knowledge, was published by Yi et al. in 2019 in which 25 patients with spinal cord‐GBM were involved (Yi et al., 2019). In this work, 10 patients with SCG were recruited. Poor expression of EMX1 was identified in the SCG tissue samples, and the expression was further decreased as the tumor grade increases, indicating the potential involvement of EMX1 downregulation in the development of SCG. Downregulation of EMX1/EMX2 genes has been documented to be correlated with the development of several epithelial‐originated solid tumors, such as gastric cancer (Li et al., 2012), lung cancer (Kim et al., 2011; Okamoto et al., 2011), endometrial cancer (Daftary & Taylor, 2004), and colorectal cancer (Aykut et al., 2017), and restoration of these genes suppressed cancer progression. Moreover, aberrant expression of EMX2 has been reported to be correlated with the development of cancer originated from melanocytes (a derivative of the neural crest), including melanoma (Bordogna et al., 2005) and GBM (Monnier et al., 2018). EMX2 reduced G1/S cell cycle arrest to block proliferation of GBM U87 cell lines (Monnier et al., 2018). Likewise, a study by Falcone et al. suggested that EMX2 suppressed GBM cell lines growth in vitro and in vivo (Falcone et al., 2016). To validate the role of EMX1 in SCG, altered expression of EMX1 was introduced in the extracted SCG cells. Overexpression of EMX1 significantly suppressed SCG cell proliferation, migration, and invasion in vitro as well as the growth and metastasis of xenograft tumors in vivo, whereas downregulation of EMX1 led to inverse trends in SCG cells. This body of evidence suggested that EMX1 downregulation is possibly correlated with the disease progression of SCG. The study by Falcone et al. indicated that the EMX2 activity relied on the modulation of diverse malignancy‐related genes (Falcone et al., 2016). In this paper, we focused on the downstream molecules of EMX1, as a transcription factor.

Later, we obtained via the bioinformatics systems (hTFtarget and Cistrome Data Browser) that WASF2 is a candidate target of EMX1, and the binding between EMX1 and WASF2 promoter was then validated through luciferase and ChIP‐qPCR assays. In line, an inverse correlation between WASF2 and EMX1 was identified in the collected SCG cells. WASF2 plays a critical role in lamellipodium formation, and the Rac1/WASF2 pathway regulates elongated movement (Yan et al., 2003). In some epithelial‐originated tumors, activation of the Rac‐WASF2 pathway has been indicated to be correlated with the migration, invasion, and drug resistance of cancer cells (Taniuchi et al., 2018; Wang et al., 2018). WASF2 has been reported to be essential for the migration and invasion of mouse melanoma cells (Kurisu et al., 2005). Moreover, inhibition of the Rac1‐WASF2 axis has been correlated with the radiosensitivity of the human glioma U251 cells (Zhou et al., 2016). Of note, in the present study, restoration of WASF2 in SCG cells blocked the role of EMX1 and rescued the cell growth and invasiveness in vitro, as well as the tumor growth and metastasis in vivo, indicating that the WASF2 downregulation is possibly implicated in the tumor‐suppressing role of EMX1 in SCG.

As mentioned above, WASF2 can potentially modulate β‐catenin activity through the interaction with ACTN4 (Hayashida et al., 2005; Taniuchi et al., 2018). Moreover, WASF2‐depleted epidermis showed subnormal activity of the Wnt signaling (Cohen et al., 2019). In addition, the EMX transcription factors have been demonstrated to inhibit tumor development by suppressing the Wnt/β‐catenin pathway (Jimenez‐Garcia et al., 2021a). The aberrant activation of the Wnt/β‐catenin pathway is causative to multiple growth‐related pathologies and is frequently involved in cancer development, including glioma (Nusse & Clevers, 2017; Shahcheraghi et al., 2020). To validate if the Wnt/β‐catenin activity is regulated by the EMX1/WASF2 axis, the protein level of β‐catenin and its role in the SCG cells were examined. It was observed that the protein level of β‐catenin in cells was suppressed by EMX1 but restored by WASF2. Importantly, the Coronaridine‐mediated inhibition of β‐catenin significantly blocked the growth and motility of SCG cells, which were promoted by WASF2, both in vitro and in vivo.

## 5 CONCLUSION

Therefore, it can be concluded that EMX1 suppresses SCG cell proliferation and metastasis by inhibiting WASF2‐mediated activation of Wnt/β‐catenin. The results of this work illustrate the correlation between EMX1 expression with the progression of SCG. EMX1 and WASF2 levels should be further explored as potential clinical metrics in SCG for patient stratification and disease prognostication. Considering that the other member of the EMX family of transcription factors, EMX2, has been proposed to as a potential suppressor of GBM cells (Falcone et al., 2016; Monnier et al., 2018), and the WNT/β‐catenin pathway has been report linking to the malignant phonotype of brain glioma (Fan et al., 2021; Xiang et al., 2022; Xu et al., 2019), the EMX1/WASF2/β‐catenin possibly also can be applied to gliomas occurring in other locations such as the brain. We would like to focus on this issue in the future researches.

## CONFLICT OF INTEREST

The authors declare no conflict of interest.

## FUNDING

None.

## Data Availability

All the data generated or analyzed during this study are included in this published article.
